# PrintrLab incubator: A portable and low-cost CO_2_ incubator based on an open-source 3D printer architecture

**DOI:** 10.1371/journal.pone.0251812

**Published:** 2021-06-02

**Authors:** Arunkumar Arumugam, Cole Markham, Saurabh S. Aykar, Barbara Van Der Pol, Paula Dixon, Michelle Wu, Season Wong

**Affiliations:** 1 AI Biosciences, Inc., College Station, Texas, United States of America; 2 Iowa State University, Ames, Iowa, United States of America; 3 University of Alabama at Birmingham, Birmingham, Alabama, United States of America; Centre National de la Recherche Scientifique, FRANCE

## Abstract

Growth in open-source hardware designs combined with the decreasing cost of high-quality 3D printers have supported a resurgence of in-house custom lab equipment development. Herein, we describe a low-cost (< $400), open-source CO_2_ incubator. The system is comprised of a Raspberry Pi computer connected to a 3D printer controller board that has controls for a CO_2_ sensor, solenoid valve, heater, and thermistors. CO_2_ is supplied through the sublimation of dry ice stored inside a thermos to create a sustained 5% CO_2_ supply. The unit is controlled via G-Code commands sent by the Raspberry Pi to the controller board. In addition, we built a custom software application for remote control and used the open-source Grafana dashboard for remote monitoring. Our data show that we can maintain consistent CO_2_ and temperature levels for over three days without manual interruption. The results from our culture plates and real-time PCR indicate that our incubator performed equally well when compared to a much more expensive commercial CO_2_ incubator. We have also demonstrated that the antibiotic susceptibility assay can be performed in this low-cost CO_2_ incubator. Our work also indicates that the system can be connected to incubator chambers of various chamber volumes.

## Introduction

Biological incubators are necessary for growing many different cell types. Although basic incubators for bacterial cultures are often inexpensive and easily obtainable, incubators that are suitable for mammalian cells and some selected bacterial cultures must be able to control and regulate their internal temperature, carbon dioxide levels, and humidity. Maintaining CO_2_ levels at 5% simulates the pH of common mammalian and bacterial cell culture data [1]. This necessary feature has driven up the price of many commercial incubators to about $5,000, which is cost-prohibitive for smaller academic labs and other point-of-care settings.

Recently, our lab required a CO_2_ incubator for use in a near-patient setting away from laboratories. Although others have also reported building a low-cost incubator with a supply of CO_2_ [2,3], we developed our own solution for a small, portable, and low-cost incubator to better fill this need. Because CO_2_ incubators are a vital resource to researchers in biomedical sciences, regardless of funding or equipment access, the information we present herein about our development will be useful to others.

Our data reveals that our compact CO_2_ incubator was able to regulate temperature and keep the bacterial cultures healthy during incubation. This result can facilitate both rapid phenotype detection of *Neisseria gonorrhoeae* (*N*. *gonorrhoea*) and the subsequent test on its antibiotic susceptibility after exposure to antibiotics. Culturing of *N*. *gonorrhoeae* requires an optimal and constant supply of CO_2_. By performing bacterial culture and antibiotic susceptibility assays in our incubator with a constant and sustained 5% CO_2_ supply, we prove that our device is comparable to the expensive commercial CO_2_ incubators. [4–7]. Our approach is an extension of our efforts to build scientific tools—using low-cost 3D printers and their components—that are versatile, inexpensive, and widely available online [8,9]. Others have also used off-the-shelf electronic components, as well as desktop 3D printing and low-cost, open-source microcontroller architecture in the development of scientific devices [10–14]. Herein, we present our study, which investigated the efficacy of the PrintrLab incubator to maintain a 5% CO_2_ concentration at 37 °C for cell growth in plates and liquid culture for several days without requiring monitoring. Our work shows that the PrintrLab incubator is a low-cost (< $400), robust, and reliable tool to grow and maintain cell cultures in comparison to a much more expensive commercial-grade CO_2_ incubator. The following sections describe how we built and tested the incubator.

## Materials and methods

**Fig 1:**
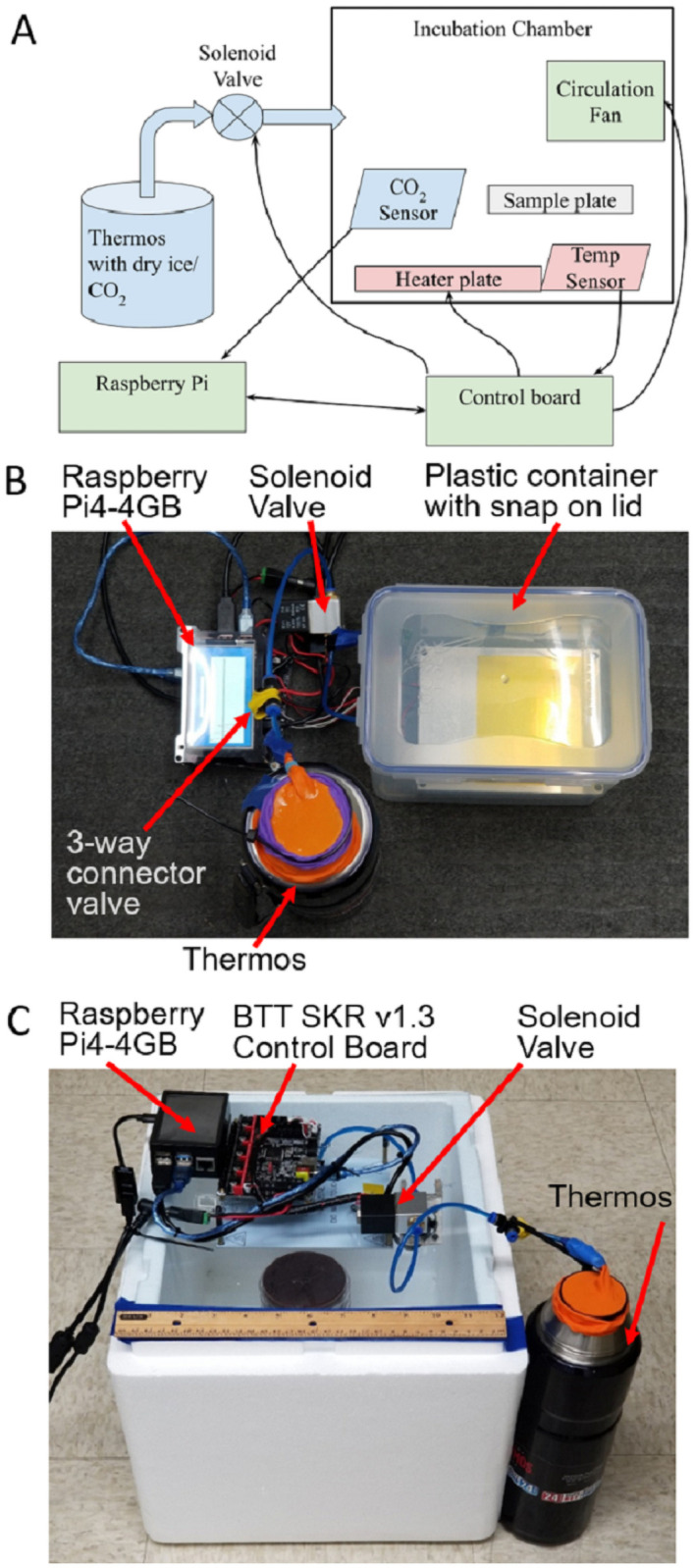
Schematic diagram and pictures of the components of device. (A) General schematic diagram of the device and setup. (B) Photo of the setup using a small plastic food storage container. (C) Larger incubator setup using Styrofoam box that can be used with the same hardware. The latex balloon (orange) and a three-way connector covered with a flexible latex band (yellow) in the CO_2_ line acted as passive venting features. This ensures a small positive pressure in the thermos to supply the CO_2,_ but without the risk of excessive pressure build up inside the thermos.

The PrintrLab incubator system presented in Fig 1 has a control system that regulates CO_2_ and temperature inside the incubator. The PrintrLab incubator is a portable incubator that consists of a leak-proof food storage container (3.9 L/431 oz. Lock and Lock brand, purchased online from Amazon.com) that houses a CO_2_ sensor (ExplorIR^®^-W 20% CO_2_ Sensor, CO2Meter.com), a 3D printer heater-bed heater, and a thermistor from a 3D printer to keep the temperature and CO_2_ concentration at levels conducive for cell growth (Fig 1). Components placed outside of the incubator include electronics, a solenoid valve, a home-made pressure-relief valve, power, and a thermos for dry ice storage. The CO_2_ concentration and chamber temperature are displayed on a screen attached to the Raspberry Pi computer unit, which can be accessed online over the internet via a Wi-Fi connection on the device [15]. The cost of the required components and the purchase links of the items are provided in Table 1. The total cost of the components is less than $400.

**Table 1:** Bill of materials.

Description	Qty	Price	Purchase Link
¼-inch DC 12V 2-way normally closed electric solenoid air valve	1	$9.30	https://amzn.to/34RfzBv
4 mm push fit (5-pack) ¼-inch connector (used 2)	1	$7.99	https://amzn.to/34SiZnN
4 mm or 5/32-inch OD polyurethane air hose pipe tube kit (10 meters)	1	$12.99	https://amzn.to/30YjpYs
Container (smaller version)	1	$17.50	https://amzn.to/2Ij1Mfo
CO_2_ sensor, ExplorIR^®^-W 20% CO2 sensor	1	$119.00	https://www.co2meter.com/collections/20-co2
Monoprice heater bed (w/ temp sensor)	1	$12.99	https://amzn.to/2DjV1Id
Circulation fan 12V	1	$9.99	https://amzn.to/2r9JcOS
Control board/BIGTREETECH SKR V1.3 32bit controller board	1	$21.99	https://amzn.to/2Em0FtZ
12V 10A power supply	1	$19.99	https://amzn.to/2ZUyb1Q
Thermos (40 oz.)	1	$21.17	https://amzn.to/371DFMP
Raspberry Pi Model 4B—4GB RAM (starter kit)	1	$99.99	https://amzn.to/33I50jK
Miuzei 4-inch IPS HDMI 800 x 480 display with touchscreen for Raspberry Pi	1	$34.99	https://amzn.to/2FWo5Xi
**Total Cost**:		**$387.89**	

### CO_2_ delivery system

**Fig 2:**
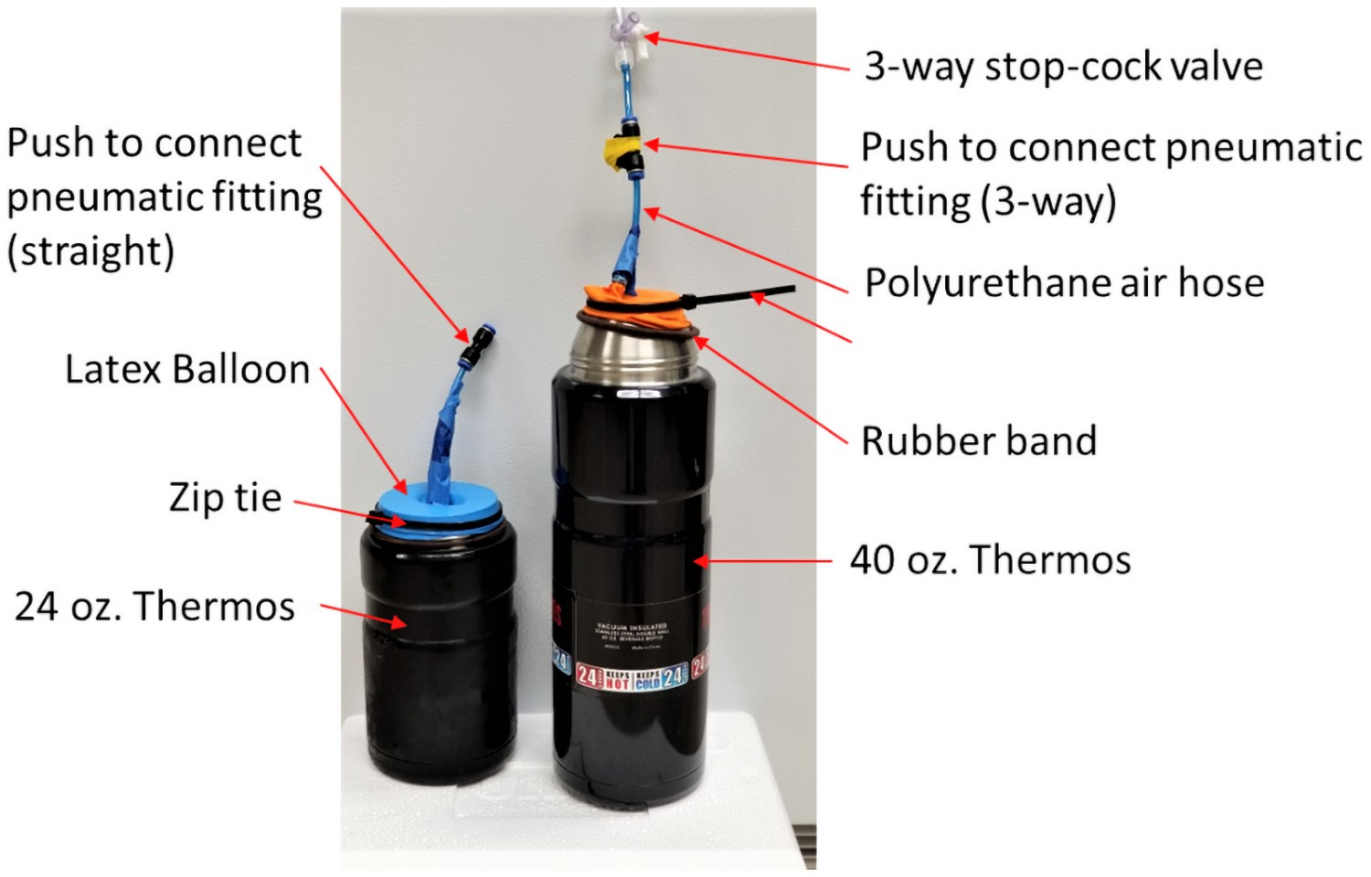
Dry ice storage containers for providing CO_2_ to the incubator. The thermos thus can store dry ice safely while providing CO_2_ to the incubator. The capacity to store more dry ice in a larger thermos allows uninterrupted use for over four days.

In our setup, we chose to use the sublimation of dry ice to supply the CO_2_ because dry ice is readily available in the United States. This approach is highly flexible and can accommodate the different incubator volumes needed. Our measurements indicated that 50 mL of CO_2_ is produced per minute from the sublimation of dry ice inside a thermos, as measured by a bubble flow meter (Optiflow 520 Digital Volumetric Flowmeter). The ability of the PrintrLab incubator to grow and possibly transport a large volume of flasks/plates with temperature control also makes it a viable option for the transport of cell therapies products over other commercially portable incubators [3]. Although storing dry ice in a capped thermos is not recommended for safety reasons, we have a simple and low-cost method to provide a lid and venting mechanism to prevent excessive pressure buildup. We capped the insulated stainless-steel thermos containers (Thermos Brand, 24 or 40 oz.) with a flexible seal made of a latex balloon that is cut at the bulb end to cover the thermos. The flexible latex material from the balloon allows the CO_2_ to expand slightly as the dry ice sublimates so that pressure does not build up in the thermos (Fig 2). Moreover, it acts as a safety pressure release since the balloon will detach from the thermos if the pressure becomes too high. During normal operation, the CO_2_ generated from sublimation leaks slightly through the balloon seal. Therefore, we have not seen the balloon inflated significantly. Using a monometer (Risepro HT-1890), we measured the pressure build up and it did not rise above 0.22 pound per square inch (PSI) (S1 Fig). A fully inflated balloon gave a PSI value less than 0.3 PSI (S2 Fig). As shown in Fig 1, we also made a passive venting system using a three-way polyurethane air hose pipe. We covered the third opening with flexible latex plastic as a relief valve. This further reduced the pressure inside the thermos to less than 0.01 PSI (S3 Fig). Overall, these features allow positive pressure to push the CO_2_ into the incubator when the solenoid valve is opened without allowing excessive pressure build up in the thermos when the solenoid valve is closed.

To regulate the delivery of CO_2_ to the chamber and achieve stable percentage readings, a solenoid valve (¼-inch DC 12V 2-Way Normally Closed Electric Solenoid Air Valve, Amazon) was placed between the thermos and the chamber, which is controlled by the system to deliver bursts of CO_2_ to the chamber based on the reading from the nondispersive infrared sensor (NDIR) CO_2_ sensor (ExplorIR^®^-W 20% CO_2_ Sensor, CO2Meter.com). Polyurethane tubing (4 mm OD) and quick-release connectors (Pneumatic 4 mm or 5/32-inch OD, 2.5 mm ID Polyurethane Air Hose Pipe Tubes) were used to connect the CO_2_ reservoir to the solenoid valve and the incubation chamber. A small hole (1/32-inch [0.7938 mm]) was drilled in the lid to provide passive venting to avoid pressure and moisture buildup. Although 0.2 to 0.4 μm air filters for incubators are commercially available and can be connected to our system to avoid bacterial or viral contaminations, we did not use them in our experiment because we mainly focused on showing growth.

### Temperature regulation

For proper incubation, the temperature in the chamber should be maintained at approximately 37 °C. The heat was provided by a 3D printer’s heated bed (repurposed from a MonoPrice MP Select 3D printer), which integrates a heating element and temperature sensor into an aluminum plate. A small computer fan (repurposed from a fan used to cool the 3D printing nozzle) circulated air within the chamber to equalize the temperature and gas concentration. A second thermistor was placed away from the heater bed to measure the ambient temperature in the chamber. While calibrating the device, we used the thermistor’s reading to determine an appropriate bed temperature that would provide sufficient heat to maintain the chamber’s temperature at 37 °C.

### System controller

**Fig 3:**
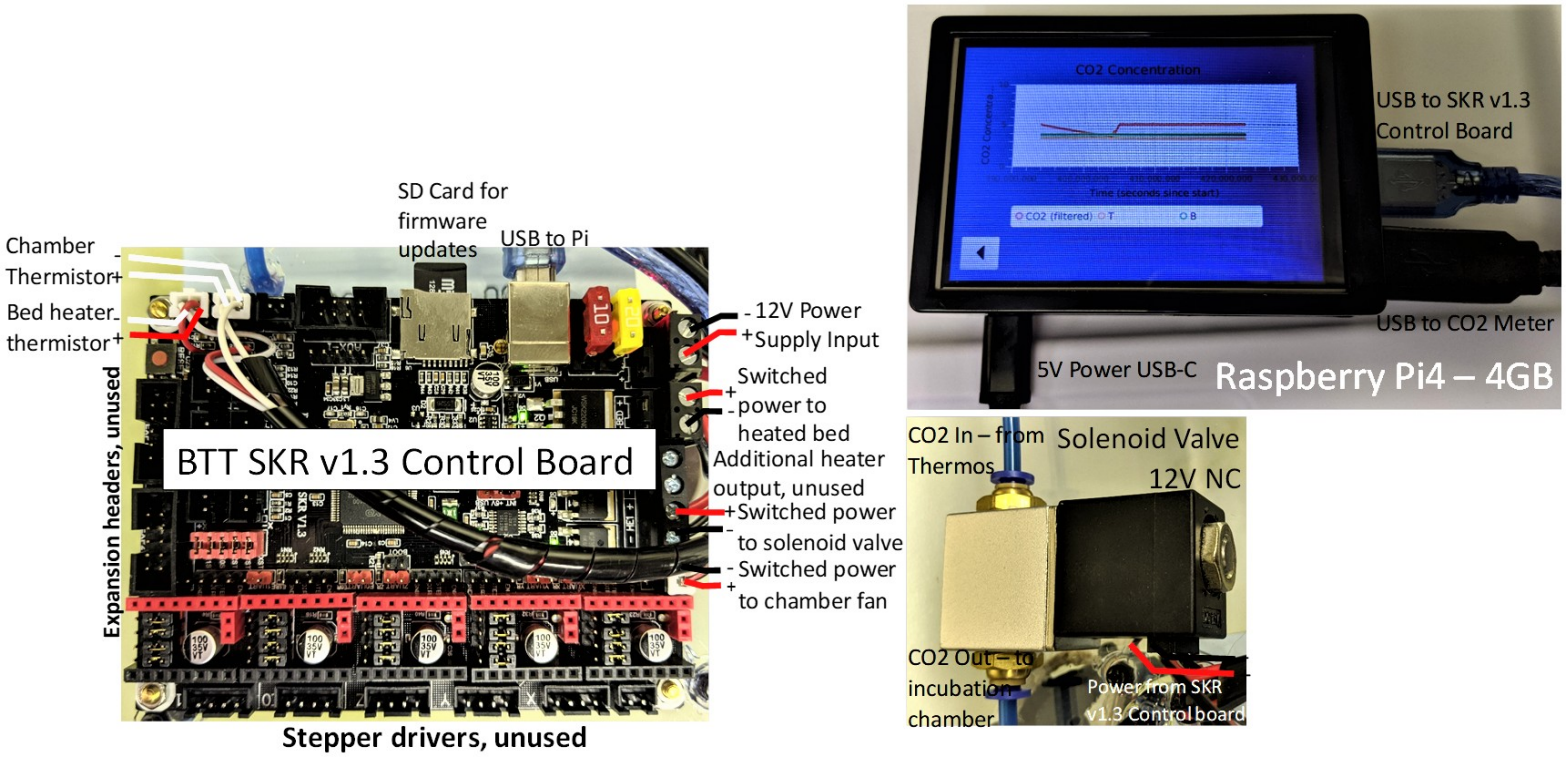
Schematic showing how the key components are connected in this 3D architecture-based device.

To control the system, we used a microcontroller board for 3D printers (BIGTREETECH 3D Printer Part SKR V1.3 32bit Control Board, Amazon.com). This control board was chosen because it has the necessary hardware to control heaters, fans, and other components. The Marlin firmware provides most of the functionality required, including fan, solenoid, and heater control. A Raspberry Pi (Model 4B) with a small touchscreen display (Miuzei 4-inch IPS HDMI 800 x 480 Display) was used to control the system and provide visualization of the temperature and CO_2_ levels. The detailed schematic in Fig 3 shows how each of the key components is connected in this 3D architecture-based device.

The Raspberry Pi is connected to both the Marlin control board and the CO_2_ meter via USB. The custom software on the Raspberry Pi uses G-code commands to control the fan, bed heater, and solenoid valve on the Marlin control board. The CO_2_ meter samples the CO_2_ concentration once per second. The sensor’s sample range is 0–20%, or 0–20,000 parts-per-million (ppm), providing sufficient coverage of the expected concentration range.

Once the setpoint is configured for the CO_2_ concentration (e.g., 5%), the software monitors the sample data and opens the valve as needed to adjust the CO_2_ level in the chamber. Initially, we employed a rudimentary control algorithm. Each time a sample was received, the software checked if the value is below the set point. If so, it checked the time since the valve was last opened. If a configured interval had elapsed, then the valve was opened for a specified amount of time (1 to 3 seconds has been tested thus far). Each of these parameters is controlled via G-code so they can be changed as needed.

**Fig 4:**
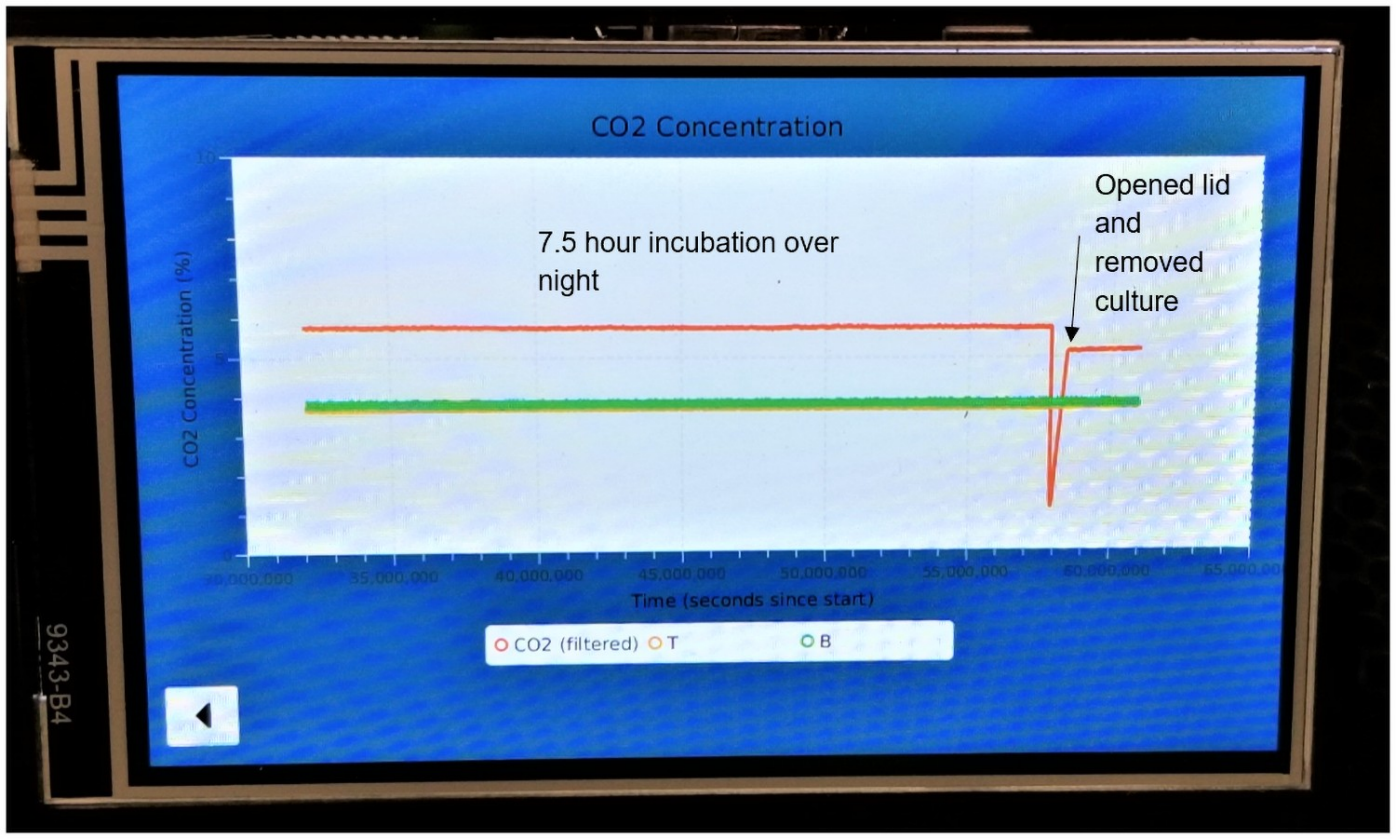
Screen display on Raspberry Pi. The CO_2_ level went up to around 6% from a 5% setting, which may have occurred due to having slightly too much CO_2_ injected every time the valve opened. The sudden drop after 7.5 hr was from the lid being opened to remove the culture. The concentration of the CO_2_ was lowered without the cells and at the duration when the valve opened (achieving 5.2% at a 5% setting). We later optimized an algorithm (sampling time and valve opening duration) to better control the concentration. Consequently, we reduced the valve opening from 3 s to 1 s so that less CO_2_ entered the incubator.

The Raspberry Pi’s touchscreen interface can be used to execute predefined scripts that start and stop the temperature and CO_2_ control. It has a graphical visualization of the temperature and CO_2_ level (Fig 4) that is updated in real time to monitor the system operation.

### Network control

The embedded control software on the Raspberry Pi includes a network interface for more advanced operations, which works with our custom Machine Control software running on a laptop or desktop computer. A user can connect to a specific embedded controller and issue commands to change the set temperature, speed, and CO_2_ control parameters.

### Data collection and remote access capability

**Fig 5:**
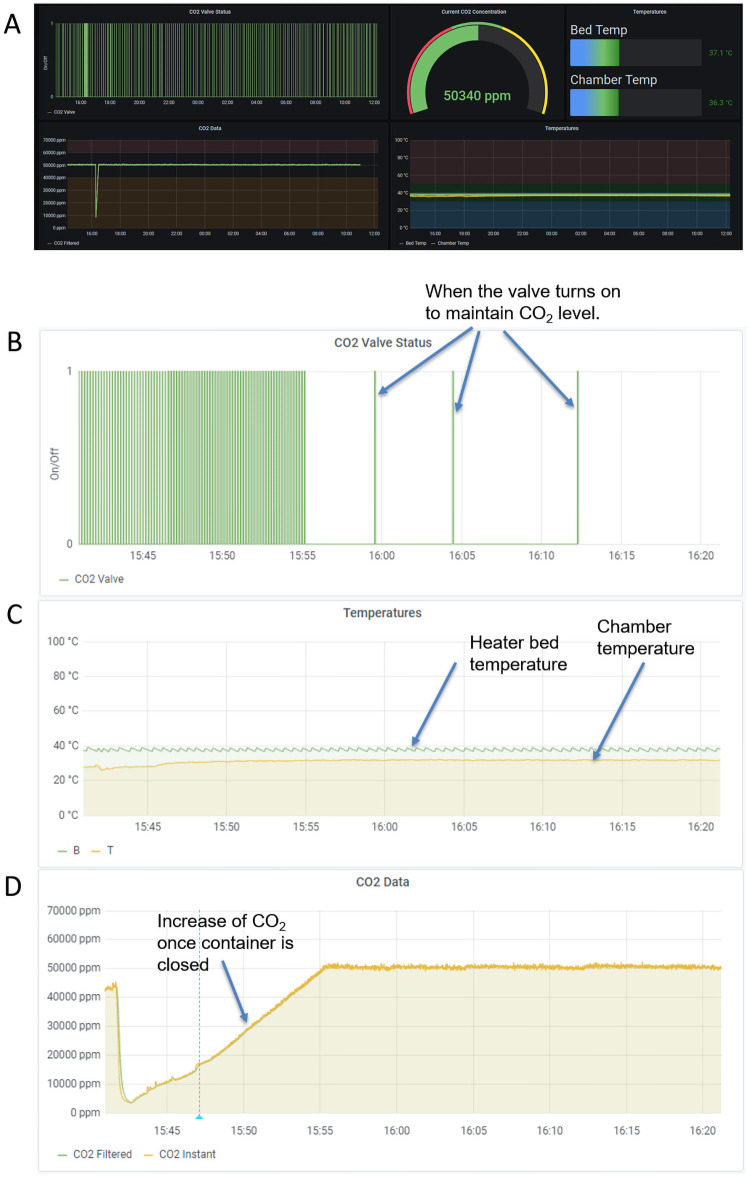
Web-based Graphana dashboard for monitoring CO_2_ levels. (A) CO_2_ concentration is stable over 16 hr. Data collected by the device included current CO_2_ concentration, the CO_2_ concentration over time, the valve’s status (on/off), bed temperature, and incubator chamber temperature, and bed/chamber temperature over time. (B) The status of the solenoid valve when the valve is opened (we programmed the valve to open for a fixed duration). (C) Temperature of the heater bed (set to 38 °C), the air temperature of the incubator, and the probe measuring the air temperature (near 36 °C as measured). (D) The CO_2_ concentration (set point of 5%) as measured by the CO_2_ sensor. The difference between CO_2_ filtered and CO_2_ instant is that the filtered values are passed through a digital low-pass filter to reduce measurement noise. This element is a configurable option in the CO_2_ Meter firmware. We used the sensor program’s default setting of 32.

Temperature and CO_2_ concentration data are uploaded in real time to a timeseries database (InfluxDB), which is linked to a Grafana dashboard to provide monitoring and analysis capabilities. Grafana is a multiplatform, open-source analytics and interactive visualization web application. It provides charts, graphs, and alerts when connected to supported data sources. Grafana allows easy sharing of the generated graphs. It is also possible to set up alerts. For instance, if the temperature or CO_2_ level is outside of the expected range, the user can be alerted. Example information displayed by Grafana is shown below in a series of graphs (Fig 5).

### Growth assessment in liquid media

A frozen stock culture of *Neisseria gonorrhoeae* (*N*. *gonorrhoeae*) (ATCC 49226) cells was used for this study. Briefly, 70 μL of frozen stock cells were diluted in 630 μL of Graver-Wade (GW) media (undiluted) which is a media for fastidious cultures [16]. These GW stock cells were further sequentially diluted 10 times (1/10×) and 100 times (1/100×). The 1/10× stock cell suspension was divided into 3 tubes, each containing 150 μL. Next, these tubes were placed in (1) a regular incubator without additional CO_2_ supply (VWR Scientific, Model 1535), (2) an AI Biosciences’ PrintrLab incubator, and (3) a commercial CO_2_ incubator (MyTemp Moni CO_2_ incubator, Benchmark Scientific, Edison, NJ). The same process was repeated with the 1/100× cell suspension, and the tubes were placed in the respective incubators. The incubators were maintained at 37 °C, and the cells were allowed to grow in these respective incubators for ~15 hr. The tubes were collected and stored at 4 °C until further analysis.

### Growth assessment using phenol red in liquid media

We used the pH indicator dye phenol red, which is commonly used in the mammalian cell culture media, to check the pH of the cell culture media and assess the growth of the cells based on their metabolic activity. The change in media color was observed. The pH drops when there’s a cell growth, and the color will change from pink to yellow.

### Growth assessment on modified Thayer Martin agar plates

The frozen stock cultures of the *N*. *gonorrhoeae* strain from ATCC were subcultured in the GW media overnight at 37 °C with a 5% CO_2_ supplementation. Thayer-Martin agar (or Thayer-Martin medium/VPN agar) is a Mueller-Hinton agar with 5% chocolate sheep blood and antibiotics. It is used for culturing and primarily isolating the pathogenic *Neisseria* bacteria, including *Neisseria gonorrhoeae* and *Neisseria meningitidis* since the medium inhibits the growth of most other microorganisms. Modified Thayer Martin agar plates (Hardy Diagnostics, Santa Maria, CA) stored at 4 °C were warmed up to room temperature. Then, 50 μL of the overnight culture cells were added and spread over the plate using a sterile L-spreader. The plates were then placed in the incubator at 37 °C in a regulated 5% CO_2_ environment and the others in the regular incubator at 37 °C without any CO_2_. The plates were incubated for 24 hr and observed for colony growth.

### Serial dilution of *N*. *gonorrhoeae* culture

The *N*. *gonorrhoeae* cells were serially diluted and plated on modified Thayer Martin agar plates for isolation and enumeration of the single colonies. The frozen stock of *N*. *gonorrhoeae* cells was cultured for ~20 hr in GW media. These cells are used as a stock for the serial dilution, and 30 μL of these stock cells were added to 270 μL of GW media in the first dilution (10-fold dilution), and this step was repeated serially for four more dilutions with GW media. The stock and diluted cells (100 μL) were plated on modified Thayer Martin agar plates and incubated in the PrintrLab incubator for 24 hr. The plates were removed from the incubator and the *N*. *gonorrhoeae* cell colonies formed in the plates were imaged using a cell phone camera.

### Real-time PCR test for growth

The growth of the *N*. *gonorrhoeae* cells in different incubators was checked by analyzing the presence of the *N*. *gonorrhoeae opa* gene through real-time PCR. The *opa* gene and the primers used in PCR of this work were commonly found in literatures to identify *N*. *gonorrhoeae*. The sequence of the primers and probes were given in Table 2. The TaqPath master mix (Thermo Fisher Scientific, Waltham, MA) containing a primer and probe set targeting the *N*. *gonorrhoeae opa* gene was added with 2 μL of retrieved cell suspension from different incubators after 15 hr of incubation [17]. Cells collected before being placed in the incubators were used as a 0 hr control. The PCR amplification was performed in a real-time thermal cycler (CFX-96, Bio-Rad, Hercules, CA), with 5 minutes of initial activation and 50 cycles of PCR with 10 seconds of denaturation at 95 °C and 20 seconds of annealing and extension at 60 °C.

**Table 2:** Primer and probe sequences.

Primers	Sequence (5’– 3’)	Annealing temperature	Product size
opa Forward	5’-TTGAAACACCGCCCGGAA-3’	60 °C	90 bp
opa Reverse	5’-TTTCGGCTCCTTATTCGGTTTAA-3’		
opa FAM Probe	5’-CCGATATAATC+CGTC+CTTCAA+CATCAG-3’		
16s rRNA Forward	5’- ACTGCGTTCTGAACTGGGTG -3’	60 °C	254 bp
16s rRNA Reverse	5’- GGCGGTCAATTTCACGCG -3’		

### Real-time PCR test for metabolic activity

To analyze the active cell growth, we measured the expression of 16s rRNA. Two μL of the cell suspension were directly added into the TB Green RT-PCR reaction mix (Takara Bio USA, Mountain View, CA) and 16s rRNA primers (Table 2) [18]. The thermal cycling was performed using the Bio-Rad thermal cycler, and the Ct values were plotted and compared. The reverse transcription step was carried out at 42 °C for 5 minutes and 10 seconds of polymerase activation at 95 °C. The PCR amplification step was performed by 5 seconds of denaturation at 95 °C and 30 seconds of annealing/extension at 60 °C for 50 cycles. The results were analyzed and compared with the sample collected and stored at the 0 hr control.

### Antibiotic susceptibility test

The frozen stock cultures of the *N*. *gonorrhoeae* strain from ATCC 49226 (ATCC) and a clinical isolate from the University of Alabama at Birmingham (UAB) were subcultured in the GW media overnight at 37 °C with 5% CO_2_ supplementation. The ATCC strain is susceptible to common antibiotics. The *N*. *Gonorrhoeae* clinical isolate (UAB 0701–22) was used in this study. This strain was isolated by Dr. Van Der Pol’s group at the University of Alabama at Birmingham. This isolate is resistant to ciprofloxacin. The antibiotic susceptibility test performed at the clinical laboratory revealed that the effective concentration of common antibiotics, such as penicillin, is ~4 μg/mL; ciprofloxacin is ~16 μg/mL; azithromycin is ~1 μg/mL; and tetracycline is ~2 μg/mL for the UAB clinical isolate. Bacterial suspension from ATCC and UAB were prepared in the GW media and used for inoculating the plates. An amount of 100 μL of the cell suspension was placed on the modified Thayer-Martin agar plate and cell spreader. The plates were allowed to dry for ~15 minutes. Ciprofloxacin (Ref # 412310) and tetracycline (Ref # 412470) Etest strips (bioMerieux, Durham, NC) were placed on each side of the plate on the agar surface. Following 24 h of incubation at 37 °C and 5% CO_2_ in the PrintrLab incubator, the minimum inhibitory concentrations (MICs) were determined by reading the lowest antibiotic concentration that inhibited growth.

## Results and discussion

### PrintrLab incubator characteristics

**Fig 6:**
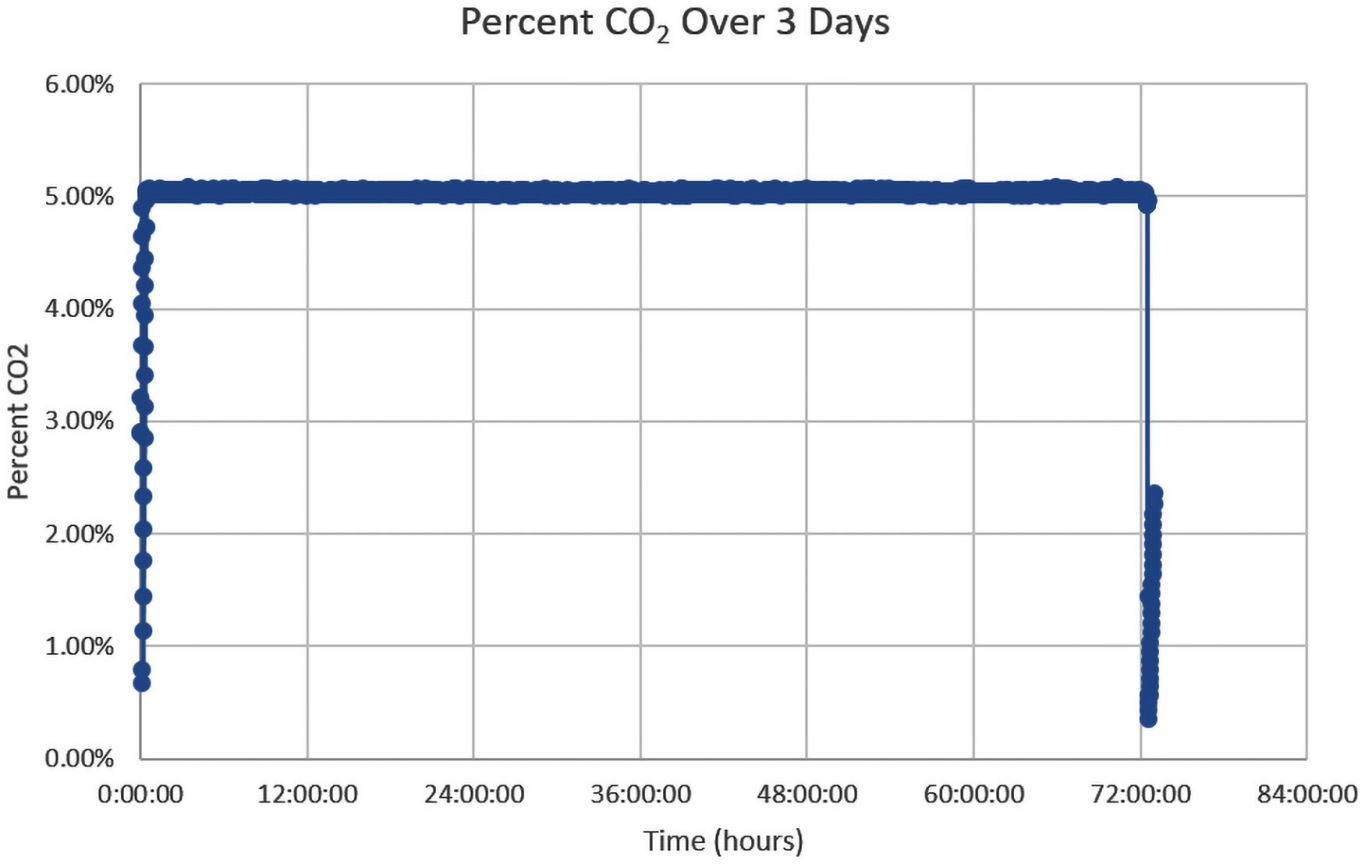
The culture conditions in the growth chamber reached equilibrium shortly after system start-up and stabilize within and between individual long-term experiments. Changes in CO_2_ concentration within the growth chamber over three days are shown. A consistent 5% CO_2_ level was maintained by the dry ice in a thermos set up without any human intervention. The rise and drop were due to adding the dry ice into the thermos at the beginning and opening the container lid after 3 days.

To assess the time required for reaching equilibrium in the PrintrLab incubator after system start-up and the stability of culturing conditions during long-term operations, we set the temperature and CO_2_ concentration to standard conditions of 37 °C and 5% CO_2_, and data points of all the sensors were recorded once per second for > 48 hr. Prior to the experiment, a Kimwipe^™^ paper wet with 3 mL of PCR-grade water was placed inside the incubator as a simple and low-cost way to supply the moisture needed. Fig 6 shows the representative time courses of CO_2_ concentration.

We note that the CO_2_ inside the cell incubator reaches a steady-state oscillation around its set point of 5%, approximately 15 minutes after system start-up. We sampled a 24 hr period during the three days, and the CO_2_ can be maintained steadily between 5.01 and 5.08%, with a standard deviation of 0.00012. During the same period, the temperature inside the incubator can be maintained steadily +/− 0.5 degrees.

Because humidification is achieved passively via evaporation from a water reservoir, the humidity inside the incubator increases slowly. The humidity reached over 99%, and the Kimwipe^™^ paper remained wet after three days of incubation. The culture conditions reach equilibrium within 20 minutes upon system start-up, so our device does not require long preparatory lead time. To speed up the building up of CO_2_ quickly rather than waiting for the CO_2_ to slowly fill up the chamber, a small chunk of dry ice (e.g., 1 gram) can be put into the chamber to allow its quick sublimation to generate CO_2_. We removed the dry ice piece as soon as we noticed that the CO_2_ concentration went above 4%. We then let the natural sublimation of CO_2_ in the thermos slowly raise the level to 5%.

In our setup, the size of the thermos and the amount of dry ice added determines how long the CO_2_ supply can last. The thermal insulation quality of the thermos also influences how fast the CO_2_ sublimates. Using a bubble flowmeter (Humonic Optiflow 520 Digital Volumetric Flowmeter), we measured that our setup has a ~ 50 mL/min flow rate. This amount of CO_2_ is more than enough to supply chambers larger than the one we tested.

We also used a digital scale (Cen-Tech Brand, Harbor Freight) to measure the amount of dry ice that sublimated when placed inside the thermoses used. Regardless of the capability, the CO_2_ measured between 150 and 190 grams. In a 40 oz. thermos, we placed 900 grams of dry ice, and it lasted well over four days, far longer than what we needed to culture our samples.

### Use of the PrintrLab incubator for *N*. *gonorrhoeae* colony formation

**Fig 7:**
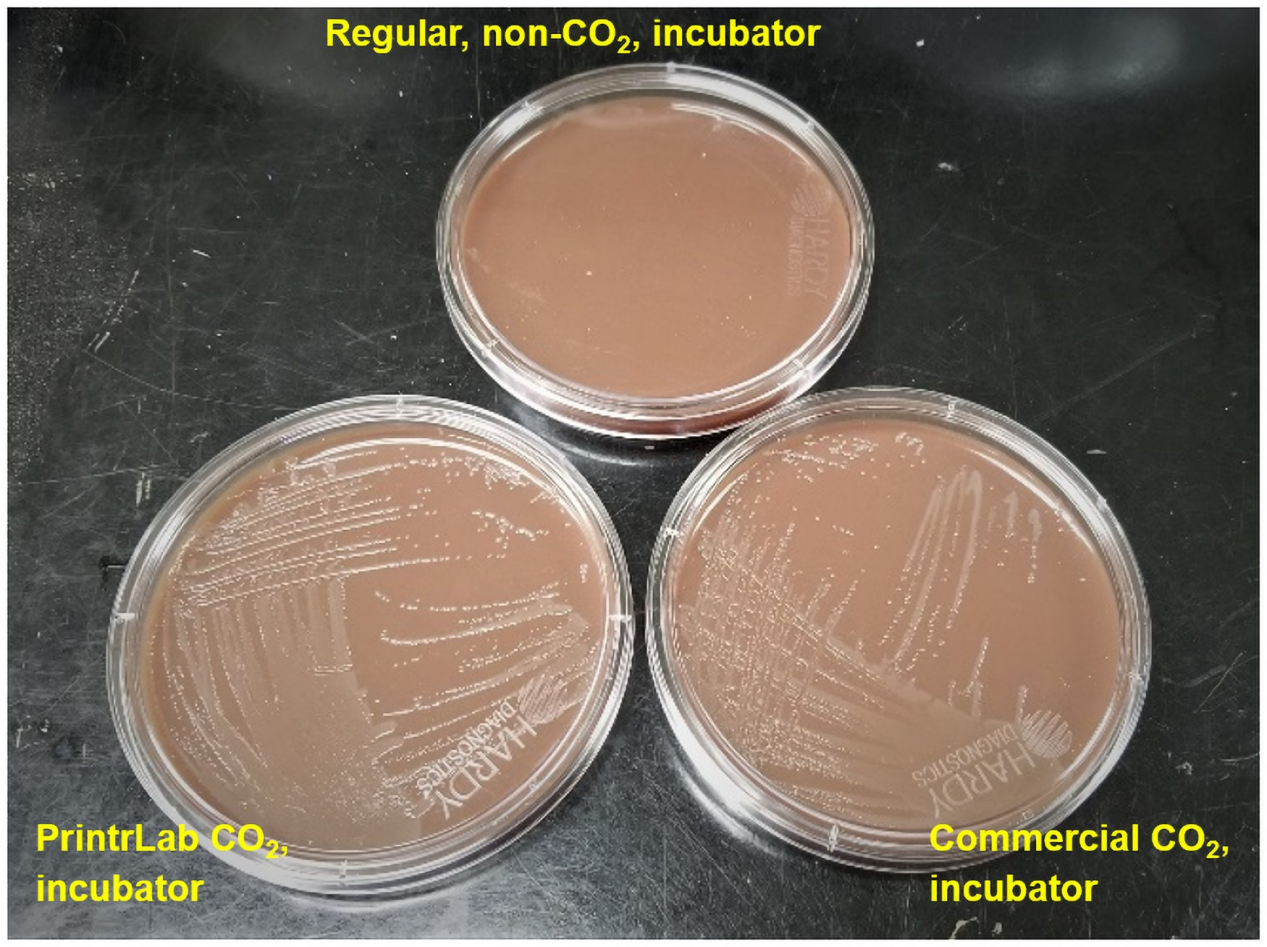
Picture of modified Thayer Martin agar plate incubated in three experimental conditions. As shown in the figure, agar plates incubated in the regular non-CO_2_ incubator (top) did not have any bacterial colonies, whereas the commercial CO_2_ incubator (right) and the PrintrLab incubator (left), with CO_2_ supplied from dry ice stored in a thermos, both had bacterial colonies.

To test the functionality of the PrintrLab incubator, we used the growth of *N*. *gonorrhoeae* on Thayer-Martin agar as an example to demonstrate its ability to incubate a bacterial culture [7]. The analysis of the growth of *N*. *gonorrhoeae* cell colonies on the modified Thayer-Martin agar plate shows that plates incubated in the incubator with a 5% regulated CO_2_ environment and 37 °C temperature showed significant colony growth on the surface (Fig 7), whereas the plates incubated at 37 °C in a regular incubator without added CO_2_ did not show any colonies. The plate stored inside a commercial CO_2_ incubator shows similar growth when compared to the one stored in the PrintrLab incubator. This indicates that the PrintrLab incubator provides suitable conditions for the growth of the *N*. *gonorrhoeae* cells.

### The serial dilution and isolation of single colonies using Printrlab incubator

The serial dilution assay was carried out to isolate and enumerate the single colonies of *N*. *gonorrhoeae* cells. The identification of colony-forming units in the sample is an indirect and semiquantitative measure of the cell viability.

**Fig 8:**
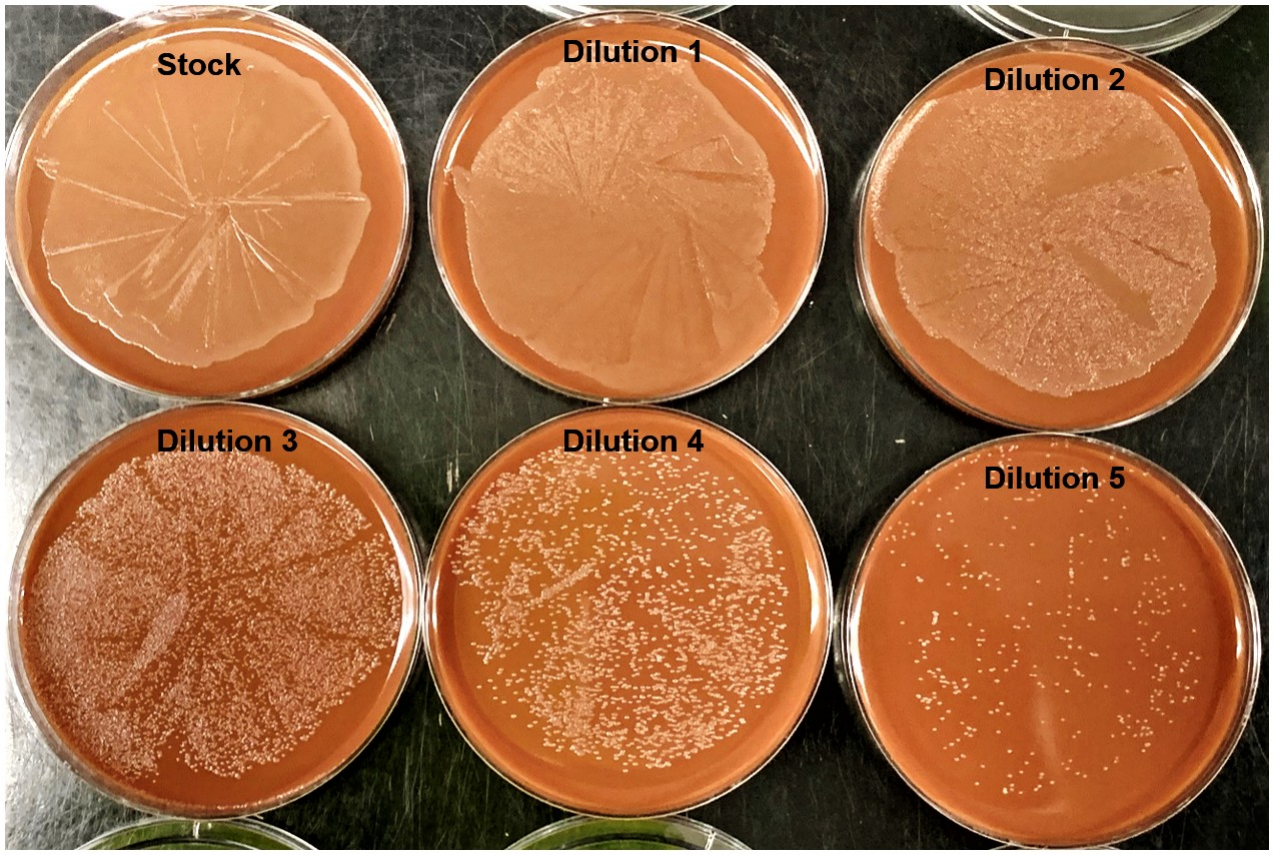
Picture of modified Thayer Martin agar plate with colonies from serially diluted *N*. *gonorrhoeae* cells. The serially diluted *N*. *gonorrhoeae* cells were incubated in the PrintrLab incubator for ~24 hr, with 5% CO_2_ supplied from dry ice stored in a thermos. The serial dilution was able to isolate and quantify the single colonies using the PrintrLab incubator.

The plates incubated at a 5% CO_2_ environment support the growth of the colonies and display approximately tenfold low colony-forming units with an increase in dilution (Fig 8). The stock and dilutions 1 (10^1^) and 2 (10^2^) displayed nearly 100% confluent growth on the plate. Dilutions 3 (10^3^) and 4 (10^4^) showed some reduction in the number of colonies, and dilution 4 is less dense than dilution 3. Dilution 5 (10^5^) showed a greater reduction in the colony number compared to the previous dilutions. The colonies were well-isolated single colonies and were easy to count manually. The isolated single colonies were obtained by plating 10^5^ dilution for manual counting (Fig 8). These results indicate that the PrintrLab incubator supports the growth and colony formation of the *N*. *gonorrhoeae* cells, which is a necessary step in the identification (by observing the morphology of the colonies), isolation, viability testing, and semi-quantification of *N*. *gonorrhoeae* in clinical specimens [19].

### Effect of CO_2_ on *N*. *gonorrhoeae* cell growth as measured by phenol red indicator dye and real-time PCR results

**Fig 9:**
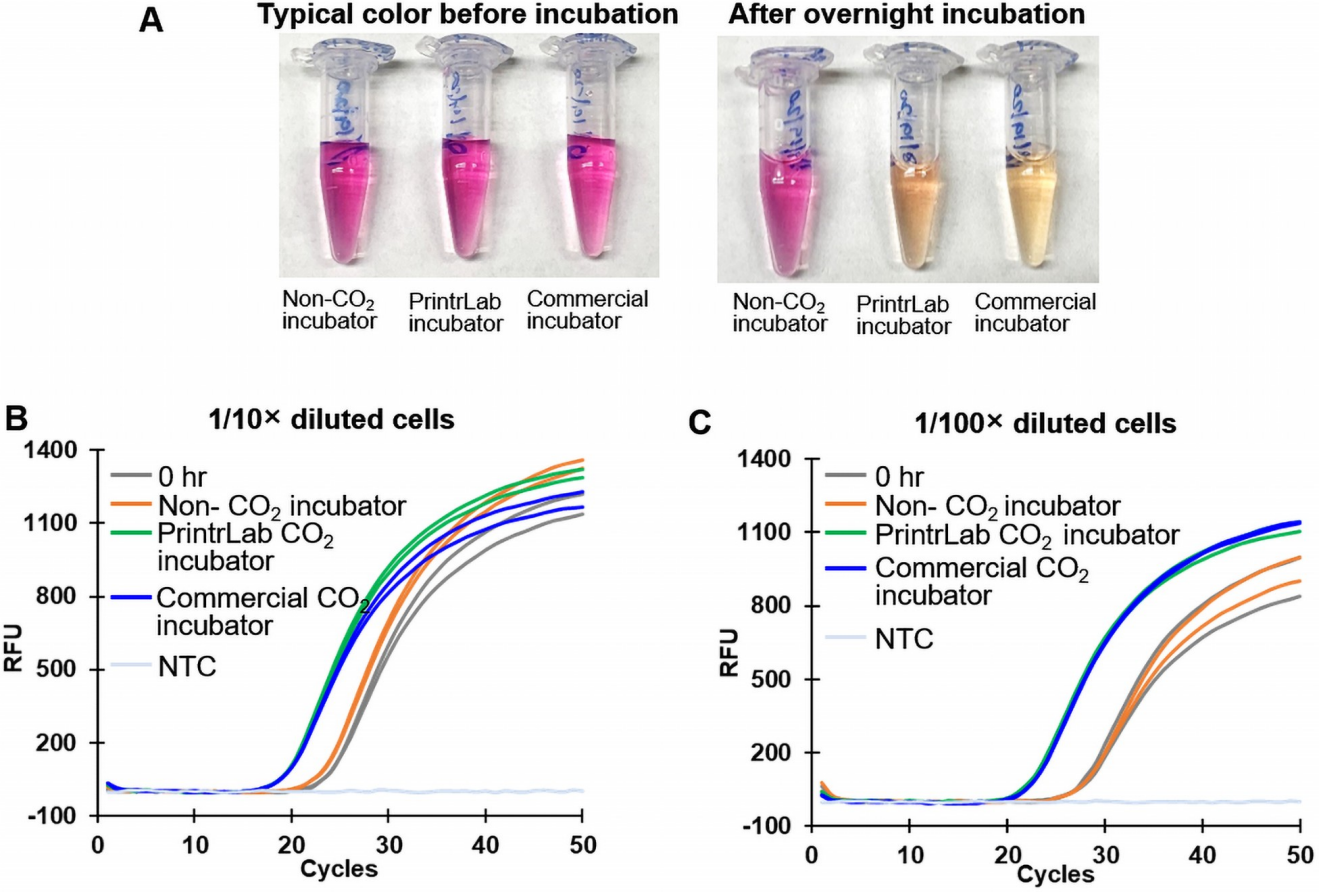
ATCC *N*. *gonorrhoeae* cells cultivated in 5% CO_2_ incubators proliferated well in 15 hr compared to the incubator providing atmospheric air without CO_2_ supplementation. (A) Photos showing the phenol red-added media’s color change after overnight culture. The color change from pink to yellow in both the PrintrLab and commercial incubators suggests that bacterial growth in these two tubes were substantially higher than in the non-CO_2_-supplied incubator. Such observation can be confirmed by real-time PCR. Duplicate samples were taken from each tube for real-time PCR. (B) 1/10× diluted cells cultured in the incubators. (C) 1/100× diluted cells cultured in the incubators. As seen by the real-time PCR curves, cell growth led to lower Ct values. Cells placed in the nonregulated incubator showed little growth when compared to the sample at 0 hr incubation. Both the 1/10× and 1/100× dilution of cells showed the same trend.

The cells were grown in the media containing the pH indicator phenol red and placed in these three incubators. After 15 hr of culturing, the media color did not change in the non-CO_2_ supplemented incubator, but there was a color change (from pink to yellow) in the cultured media stored in the two other 5% CO_2_ supplementing incubators (Fig 9A). The color change from pink to yellow indicates that the media became acidic due to the secretion of metabolites from proliferating cells. Therefore, the color change is an indication that the cells were proliferating well in the PrintrLab incubator and commercial incubator in comparison to the non-CO_2_ supplementing incubator. We used real-time PCR amplification of the *opa* gene to check the growth difference revealed by the use of phenol red medium when the *N*. *gonorrhoeae* cells were placed in different incubators. Each tube was sampled twice for real-time PCR. The results revealed that the 1/10× diluted cells placed in the incubator without a CO_2_ supply did not show much growth after a 15 hr incubation period when compared to a 0 hr control. The threshold cycle (Ct) value of the 0 hr control was 24.0, and the 15 hr incubation in the non-CO_2_ incubator was 23.19 (Fig 9B). On the other hand, the cells cultivated inside the PrintrLab incubator with 5% CO_2_ supplementation and the commercial CO_2_ incubator showed excellent and similar growth based on the Ct values of 19.5 and 19.7, respectively (Fig 9B). We also observed the same trend in the 1/100× diluted cells grown in these conditions. The average Ct of cells incubated for 15 hr was 28.1 in an incubator without a CO_2_ supply, 22.5 for the PrintrLab incubator, 22.7 for the commercial incubator, and 28.0 for the 0 hr control (Fig 9C). This result indicates that CO_2_ is required for the growth of *N*. *gonorrhoeae* cells, and the PrintrLab incubator supplies an appropriate amount of CO_2_ to support the growth of *N*. *gonorrhoeae* cells in a culture.

### Effect of CO_2_ on the metabolic activity of *N*. *gonorrhoeae* cells

**Fig 10:**
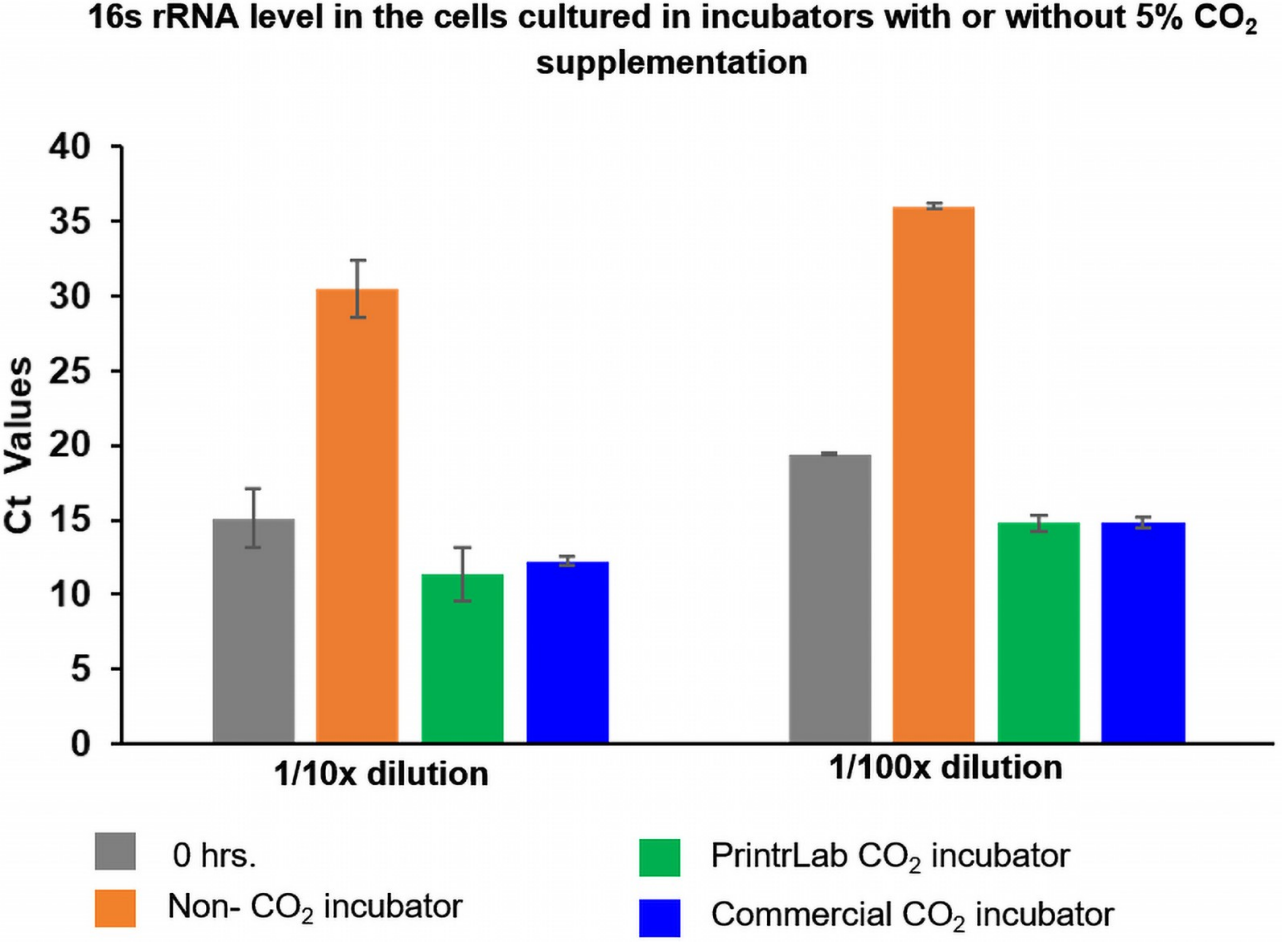
16s rRNA level in samples at two concentrations stored in a regular (non-CO_2_ supply), commercial, and PrintrLab incubator for 15 hr. The sample at time zero was used as a reference. The culture conditions in the growth chamber reached equilibrium shortly upon system start-up and were stable within individual long-term experiments. The error bars of the plots showed the standard deviation based on samples taken from two duplicating tubes.

As an indirect measure of metabolic activity, we analyzed the expression level of 16s rRNA in the cells cultured in different incubators. The results reveal that the cells cultured in the non-CO_2_ supplement incubator showed a drastically reduced expression of 16s rRNA (Ct value of 30.4 and 36.0 for 1/10× and 1/100×, respectively) compared to the respective 0 hr time point of the 1/10× (Ct–15.1) and 1/100× (Ct–19.4) dilutions (Fig 10). The cells grown in the incubators with controlled CO_2_ supplementation showed better expressions of 16s rRNA. The PrintrLab incubator cells showed 11.3 and 14.8 Ct values for 1/10× and 1/100× diluted cells, and the commercial incubator cells showed 12.2 for 1/10× dilution and 14.8 for 1/100× dilution of cells (Fig 10). Collectively, the data suggest that the cells cultivated in the 5% CO_2_-controlled environment showed more metabolic activity (measured indirectly) and better growth than the incubator without CO_2_ supplementation.

**Fig 11:**
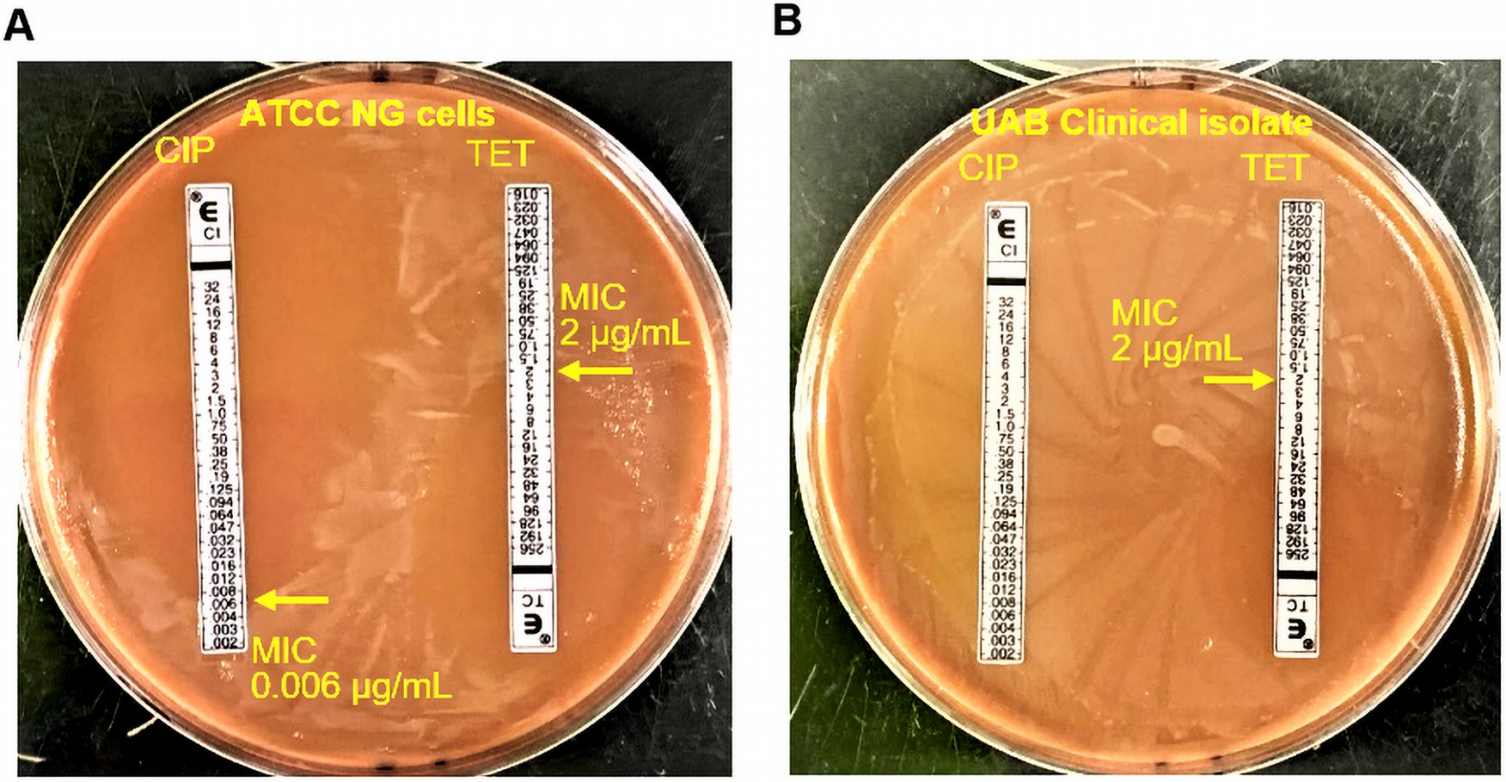
Antibiotic susceptibility test performed using Etest strips. Representative pictures of Etest for the determination of antibiotic susceptibility of ATCC *N*. *gonorrhoeae* cells (A) and UAB clinical isolate (B) with ciprofloxacin (CIP) and tetracycline (TET) Etest strips. The UAB clinical isolate was resistant to ciprofloxacin, confirming results from the previous characterization.

We next performed an experiment to show that the PrintrLab incubator can be used for performing antibiotic susceptibility test with Etest strips. According to the Etest result shown in Fig 11, the MIC for the ATCC *N*. *gonorrhoeae* cells is 0.006 μg/mL of ciprofloxacin and 2 μg/mL of tetracycline. For the clinical isolate received from UAB, ciprofloxacin did not show any inhibition effect on the cell growth, but ~ 2 μg/mL of tetracycline inhibited the growth of the cells (Fig 11). The Etest result confirmed that the UAB clinical isolate, previously characterized as ciprofloxacin resistant, displayed similar resistance to ciprofloxacin in our experiment performed using the PrintrLab incubator. The susceptible ATCC cells showed an expected susceptibility range in experiments carried out in the low-cost PrintrLab incubator.

### Limitation of this work

We understand that a full-feature incubator requires the perfect synergy between conditions such as temperature, humidity, and CO_2_/O_2_ concentrations in the chamber. Each condition can affect the cells if not monitored carefully. While our PrintrLab incubator has the ability to maintain CO_2_ and temperature accurately and deliver condition statuses in real-time, it does not yet provide humidity and O_2_ controls. We also have not tested the use of the incubator for mammalian cell culture.

## Conclusions

To overcome the limitations of commercially available products suitable for portable and low-cost (<$400) CO_2_ incubation, we have developed a platform with a “Makers” approach through the utilization of 3D printer architecture, which allowed us to build a device that is easy to use, customizable, robust, and affordable to a broad range of educational institutions and research labs. In this paper, we demonstrated the viability of building a low-cost, portable CO_2_ incubator. The temperature was maintained steadily using heater bed components. By using dry ice stored inside a thermos as a source of CO_2_, we were able to maintain a 5% CO_2_ level and 37 °C for over three days. We have demonstrated *N*. *gonorrhoeae* cell growth inside our 5% CO_2_ incubator when the same culture did not show growth in a regular, non-CO_2_ incubator. We also used real-time PCR to validate the cell growth in the GW liquid media. The results showed that minimal growth was observed from the sample incubated at 37 °C without the presence of CO_2_. On the other hand, our CO_2_ incubator provided the correct environment for cell growth, as indicated by the reduction of the threshold Ct of basal gene expression of *N*. *gonorrhoeae* in the real-time PCR reaction. More importantly, the growth of the cells in our incubator is comparable to the cell growth found in a commercially available CO_2_ incubator.

Moreover, the percentage of CO_2_ concentration and the temperature of the incubator were readily available via a remote-access website or on the LCD display unit of the device. In addition to cell growth and transport ability, we plan to add a camera and imaging capability for time-lapse imaging of samples. In short, our low-cost approach can be made into a conversion kit that will enable other users to build and maintain a small CO_2_ incubator at a cost that is significantly lower than any commercial device.

## Supporting information

S1 Fig Pressure inside the balloon-covered thermos that contained dry ice. Only a low pressure was measured (0.219 PSI) and the balloon would slip off if pressure continued to increase.
(PDF)

S2 Fig Pressure inside an inflated latex balloon. A well-inflated balloon only had a pressure of 0.273 PSI as measured by the manometer.
(PDF)

S3 Fig Pressure inside the balloon-covered thermos that’s also connected to a second relief valve. This reduced the pressure in the system to a very low number (e.g., 0.004 PSI). This small pressure was enough to supply CO_2_ into the incubator when the solenoid valve was opened.
(PDF)
