# Supplementary material for: PrintrLab incubator: A portable and low-cost CO2 incubator based on an open-source 3D printer architecture
Source: PLoS One. 2021 Jun 2;16(6):e0251812. doi: 10.1371/journal.pone.0251812 (PMC8172042; doi:10.1371/journal.pone.0251812)
Supplement: S1 Fig — Only a low pressure was measured (0.219 PSI) and the balloon would slip off if pressure continued to increase. (PDF) [file pone.0251812.s001.pdf]

S1 Fig.

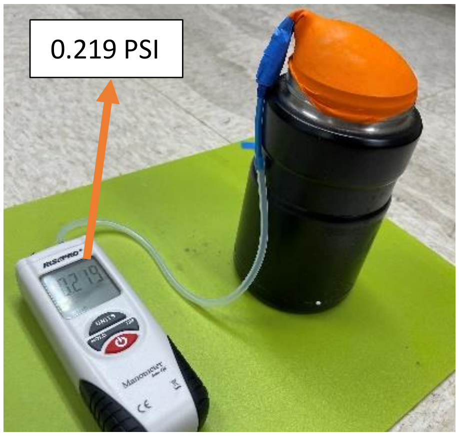

S1 Fig. Measuring the pressure buildup with only a balloon covering the thermos (0.219 PSI). Balloon only inflated slightly due to dry ice sublimation.
