# Supplementary figures and images for: PrintrLab incubator: A portable and low-cost CO2 incubator based on an open-source 3D printer architecture
Source: PLoS One. 2021 Jun 2;16(6):e0251812. doi: 10.1371/journal.pone.0251812 (PMC8172042; doi:10.1371/journal.pone.0251812)

**S2 Fig.**

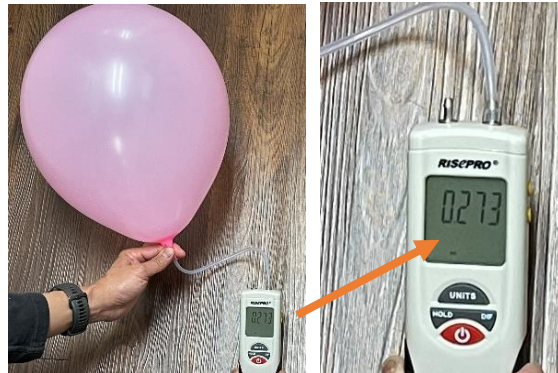

**S2 Fig.** Measuring the pressure inside a well-inflated balloon (0.273 PSI).

Supplement: S2 Fig — A well-inflated balloon only had a pressure of 0.273 PSI as measured by the manometer. (PDF) [file pone.0251812.s002.pdf]
