# Supplementary material for: PrintrLab incubator: A portable and low-cost CO2 incubator based on an open-source 3D printer architecture
Source: PLoS One. 2021 Jun 2;16(6):e0251812. doi: 10.1371/journal.pone.0251812 (PMC8172042; doi:10.1371/journal.pone.0251812)
Supplement: S3 Fig — This reduced the pressure in the system to a very low number (e.g., 0.004 PSI). This small pressure was enough to supply CO2 into the incubator when the solenoid valve was opened. (PDF) [file pone.0251812.s003.pdf]

S3 Fig.

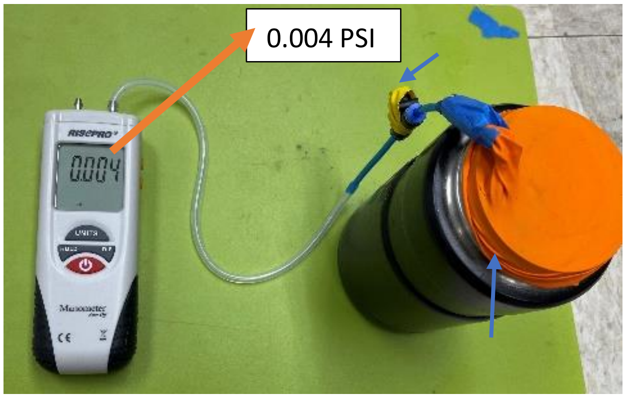

S3 Fig. Using a manometer to measuring the pressure buildup with a balloon and three-way connector connected to the dry ice-filled thermos (0.004 PSI). Blue arrows indicates where CO_2 can escape to prevent pressure build up.
